# Unveiling MRI-based structural phenotypes in temporomandibular joint osteoarthritis: implications for clinical practice and research

**DOI:** 10.1590/2177-6709.29.4.e24spe4

**Published:** 2024-09-02

**Authors:** Ricardo de Souza TESCH, Thayanne Brasil Barbosa CALCIA, Diego DE NORDENFLYCHT

**Affiliations:** 1 Centro Universitário Arthur Sá Earp Neto/Faculdade de Medicina de Petrópolis (UNIFASE/FMP).; 2 University of Andrés Bello, School of Dentistry (Santiago, Chile).

**Keywords:** Magnetic resonance imaging, Osteoarthritis, Temporomandibular joint, Temporomandibular disorders, Ressonância magnética, Osteoartrite, Articulação temporomandibular, Disfunções temporomandibulares

## Abstract

**Introduction:**

Osteoarthritis (OA) is a progressive degenerative disease characterized by the gradual degradation of cartilage, remodeling of subchondral bone, synovitis, and chronic pain. This condition impacts various large and small joints, including the temporomandibular joint (TMJ). However, addressing OA, particularly in impeding or reducing disease progression, is challenging due to its clinical and imaging heterogeneity. Authors are increasingly suggesting that this heterogeneity involves different phenotypes or subpopulations, discernible by variations in the disease’s pathophysiology and structural manifestations. Even within the TMJ, these phenotypes may display distinct clinical features, laboratory parameters, biochemical markers, and imaging criteria. Recent research has proposed MRI as a reference standard for TMJ OA, highlighting its substantial agreement with histopathological changes. MRI-based phenotypes offer a promising avenue for understanding disease progression and treatment response, potentially providing valuable insights for prognosis and treatment planning.

**Objective:**

This article introduces the ROAMES-TMJ (Rapid OsteoArthritis MRI Eligibility Score for TMJ) to assess the structural eligibility of individuals for inclusion in TMJ OA clinical trials.

## RESUMO

**Introdução:**

A osteoartrite (OA) é uma doença degenerativa progressiva caracterizada pela degradação gradual da cartilagem, remodelação do osso subcondral, sinovite e dor crônica. Essa condição afeta várias articulações grandes e pequenas, incluindo a articulação temporomandibular (ATM). No entanto, abordar a OA, particularmente na prevenção ou redução da progressão da doença, é um desafio devido à sua heterogeneidade de sua apresentação clínica e em exames de imagem. A literatura sugere cada vez mais que essa heterogeneidade envolve diferentes fenótipos ou subpopulações, discerníveis por variações na fisiopatologia e nas manifestações estruturais da doença. Mesmo dentro da ATM, esses fenótipos podem apresentar características clínicas, parâmetros laboratoriais, marcadores bioquímicos e critérios de imagem distintos. Pesquisas recentes propuseram a ressonância magnética como padrão de referência para OA da ATM, destacando sua concordância substancial com alterações histopatológicas. Os fenótipos baseados em ressonância magnética oferecem um caminho promissor para a compreensão da progressão da doença e da resposta ao tratamento, fornecendo potencialmente informações valiosas para o prognóstico e o planejamento do tratamento.

**Objetivo:**

Este artigo apresenta o ROAMES-TMJ (*Rapid OsteoArthritis MRI Eligibility Score for TMJ*) para avaliar a elegibilidade estrutural de indivíduos para inclusão em ensaios clínicos de OA de ATM.

## INTRODUCTION

The public health impact of osteoarthritis (OA) is increasingly significant, marked by a rising prevalence that makes it the most common among around a hundred forms of arthritis. This escalating prevalence brings substantial disability and economic burdens.^1^ Currently, interventions are limited to exercises, self-management programs, analgesics, and, in cases of severe and unresponsive conditions, more invasive procedures. These secondary approaches may range from minimally invasive intra-articular injections of various medications and biologically active substances to arthroscopic or open surgeries, ultimately leading to total joint replacement.^2^ Given these challenges, there is an urgent need to deepen our understanding of OA’s underlying etiology, identify effective treatments, and establish preventative strategies to alleviate pain and minimize joint damage.

Efforts to address OA, especially in impeding or reducing disease progression, face obstacles due to the heterogeneity of the condition. Formulating a universally applicable therapy for such a diverse and unselected patient population proves to be a daunting task.

OA can stem from various factors, including post-traumatic events, genetic predisposition, metabolic influences, and biomechanical issues.^3^ Furthermore, the complexity is heightened by the involvement of multiple mechanisms in pain perception associated with OA.^4^ Recognizing this heterogeneity, there is a growing consensus that OA is not a singular disease, but rather a syndrome involving the joints as an organ, encompassing multiple distinct phenotypes.^5^

As a syndrome, OA affects different large and small joints, including the temporomandibular joint (TMJ). While the knee, a large synovial joint, is one of the most frequently affected, with about 14% of the adult US population affected by knee OA,^6^ the TMJ experiences a similar incidence of cartilage ailments. However, the knee orthopedics field has greater funding and more effective end-stage treatment options, being considered as a template for the development of TMJ diagnosis and treatment strategies.^7^ This perspective forms the foundation of this narrative review, which centers on the concept of knee OA phenotypes^8^ and underscores the potential significance of defining and understanding structural phenotypes of TMJ OA through imaging.

Thus, the purpose of the present article is to introduce, for future validation, the Rapid OsteoArthritis MRI Eligibility Score for TMJ (ROAMES-TMJ), to evaluate the structural eligibility of individuals for inclusion in TMJ OA clinical trials. Through this initial narrative review, we aim to categorize individuals into distinct structural phenotypes, thereby acknowledging the diverse pathological changes associated with TMJ OA.

## THE IMPORTANCE OF TMJ OA PHENOTYPES

Pathological changes in OA can manifest in multiple joint tissues, providing a myriad of potential treatment targets. Given the multifaceted nature of tissue involvement in OA, it is improbable that a singular treatment approach will be universally effective in preventing or slowing the progression of all structural OA types. Authors increasingly propose that OA encompasses various phenotypes or subpopulations, distinguished by the disease’s pathophysiology and structural manifestations.^9^ Even in the TMJ, these phenotypes may exhibit distinct clinical features, laboratory parameters, biochemical markers, and/or imaging criteria.^10^

Traditionally, the TMJ OA field has centered around an articular cartilage disease model. However, the timing and sequence of cartilage erosive changes are still controversial. It is not clear whether the osteoarthritic degenerative disease begins in the superficial or calcified deep layers of the articular cartilage, or even subchondral bone. Thus, more recently, a bone-driven cartilage progression disease model has been proposed.^11^ However, inflammation is now acknowledged as a central aspect of OA pathology. While not always the primary initiator, inflammation is present in early OA stages and may become the driver of disease progression.^12^

The challenges faced by numerous phase II/III OA clinical trials, and the potential failure to translate short-term clinical benefits into real structural improvement may be partly attributed to difficulties in identifying patient subpopulations with structural abnormalities aligning with drug pharmacodynamic. Understanding OA phenotypes is crucial for developing targeted treatments and assessing the efficacy of disease-modifying osteoarthritis drugs. Current Diagnostic criteria for temporomandibular disorders (DC/TMD) indicates that computed tomography (CT) or cone beam CT (CB-CT) are needed for diagnosis confirmation of OA;^13^ however, recent research suggested the MRI as a reference standard for TMJ OA, given its substantial agreement with histopathological changes.^14^

In this sense, MRI-based phenotypes offer a promising avenue for comprehending disease progression and treatment response, potentially providing prognostic and treatment planning value.^15^ Synthesis of main clinical and imaging features of each OA phenotype proposed is shown in Table 1.

**Table 1:** Main clinical and imaging features of each osteoarthritis phenotype.

Phenotype	MRI	CT	USG	Signs and symptoms
Adaptive	Flattening	Flattening Focal sclerosis	X	X
Cartilage erosive	Surface irregularity	X	X	Pain during function
Hypertrophic	Osteophyte	Osteophyte	Osteophyte	Crepitus
Bone	Bone marrow lesion	Subchondral cyst	X	Orthopedic instability
Inflammatory	Effusion	X	Capsule enlargement	Pain on mouth opening

MRI = magnetic resonance imaging; CT = computed tomography; USG = ultrasonography.

## CARTILAGE EROSIVE PHENOTYPE

While MRI is effective in identifying the cartilage layer in large joints, it faces challenges in accurately detecting degenerative changes in mandibular condylar cartilage due to its extremely thin nature. This distinctive fibrocartilage, which have a thickness of 1.5 to 2 mm in the mandibular condyle and 0.5 to 1 mm in the mandibular fossa,^16^ is highly susceptible to the mechanical environment, and overloading can precipitate degenerative changes, which can be reversed by self-repair at early stages.^17^ Thus, recognizing the pivotal role of the disc in load distribution and shock absorption for safeguarding cartilage, an erosive cartilage phenotype often presents itself alongside disc displacement and/or damage, leading to widespread cartilage loss. Histological assessments classify cartilage changes into four sub-grades: intact surface, surface discontinuities, vertical fissures, and erosion, based on the depth of the zone (fibrous, proliferative, or hypertrophic) to which the degenerative processes comprise.^14^

Irreversible histological damage was found in TMJ disc displacement without reduction. In patients with clinical diagnosis of disc displacement without reduction and limited mandibular opening, about 56% of the TMJ fibrocartilage was absent or only focally present. When diagnosing degenerative joint disease, clinical diagnosis of joint crepitus confirmed by CT images shows a significant increase in these numbers, with total fibrocartilage absence in up to 30% of analyzed joints, and only focal presence in over 50%. In these cases, none of the joints exhibit intact fibrocartilage.^18^

**Figure 1:**
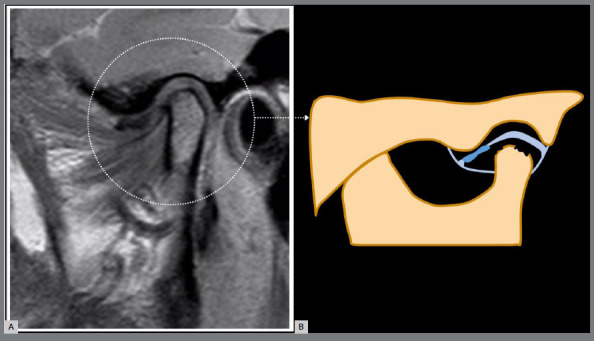
Cartilage erosive phenotype: a loss of continuity of the articular cortex of the condyle can be observed. A) Referential temporomandibular joint MRI sagittal T1-weighted image. B) Temporomandibular joint scheme. Adapted from ROAMES - Rapid OsteoArthritis MRI Eligibility Score (Source: Roemer et al.^8^, 2020).

Cartilage loss exposes the subchondral bone through condylar surface, leading to progressive bone erosion (Fig. 1). Therefore, MRI-detected condylar erosion signifies changes in articular cartilage and the adjacent cortical and subcortical bone, correlating with characteristic clinical findings such as pain, joint sounds, and deviating mandibular movement.^19^ Synovial activation, considered a secondary phenomenon related to cartilage deterioration, leads to joint effusion, demonstrated to be associated with arthralgia.^20^ While it can be present in cases without arthralgia, its frequency is directly proportional to pain scores, increasing with the intensity of pain.^21^

While examining the impact of non-surgical methods on young patients under 18 years, with TMJ-related symptoms, a notable reduction in the proportion of patients with erosive abnormalities was observed. However, despite this improvement, approximately half of these patients were unable to develop an intact cortical outline repair after a median follow-up period of 4 years.^22^ Further investigation, specifically focusing on asymptomatic patients after conservative treatment, revealed an intriguing natural course during follow-up (mean two years). Most condyles with cortical erosions evolve into an intact and continuous cortex. However, it is crucial to note that in severe TMJ OA, the discontinuous cortex type was found to be unstable, when compared to the continuous cortex type. Condyles exhibiting a discontinuous and hypodense cortex displayed an increased probability of volume reduction over time,^23^ which suggests a risk for occlusal changes.

## HYPERTROPHIC PHENOTYPE

In the realm of OA, a hypertrophic phenotype is distinguished by the presence and size of osteophytes.^8^ An osteophyte is a marginal hypertrophy characterized by sclerotic borders and the exophytic angular formation of osseous tissue arising from the surface.^24^ These fibrocartilage-capped bony outgrowths, originating from the periosteum, contribute to the complexity of OA, through cartilage degradation.^25^

The pathophysiology of osteophyte formation remains incompletely understood, prompting ongoing research endeavors. To facilitate systematic investigation, a histological osteophyte classification has been proposed, allowing for the differentiation of four distinct types based on ossification degree and the percentage of mesenchymal connective tissue.^25^ This classification, established for basic science research questions, demonstrates that osteophyte size and localization are independent of histological stages.

**Figure 2:**
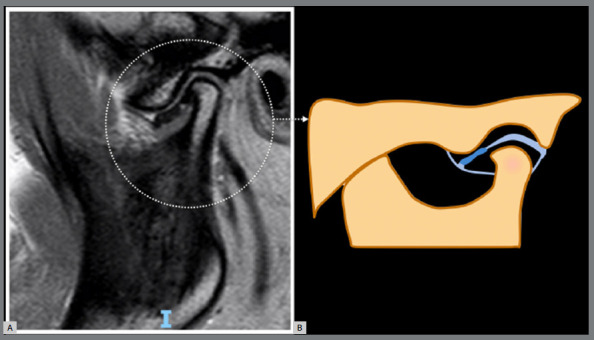
Hypertrophic phenotype: a marginal hypertrophy with sclerotic borders and exophytic angular formation of osseous tissue arising from the surface of the condyle (osteophyte) can be observed. **A**) Referential temporomandibular joint MRI sagittal proton density image. **B**) Temporomandibular joint scheme. Adapted from ROAMES - Rapid OsteoArthritis MRI Eligibility Score (Source: Roemer et al.^8^, 2020).

Histopathological interpretation of osteophytes in the TMJ is notably lacking in the literature. However, a histopathological examination of TMJ osteophytes revealed fibrocartilaginous core tissue surrounded by bone formation due to dystrophic calcification.^26^ The relationship between osteophyte formation and cartilage damage is nuanced. While osteophytes can be associated with cartilage damage, they may also occur independently, prompting discussions about whether they represent a pathological phenomenon or a functional adaptation (Fig. 2). Notably, osteophytes are common in OA and can lead to clinically relevant symptoms, but they can also be present without negative effects.^27^

Distinct periosteal and synovial skeletal progenitors have been identified as contributors to osteophyte formation in OA, suggesting potential targets for disease modification in OA treatment.^28^ Mechanically-induced osteophytes in the rat knee highlight the role of moderate trauma to the periosteal layer in osteophyte development.^29^ Furthermore, in TMJ-related studies, a statistically significant association between osteophytes and disc displacement has been observed, especially among cases of disc displacement without reduction.^30^ A nine-times greater likelihood of osteophyte occurrence was observed in cases of anterior disc displacement without reduction (ADDwoR), whereas a lower OR for their occurrence (OR=2.96) was observed in cases with reduction (ADDwR).^31^ This underscores the complex interplay between clinical factors, joint changes, and osteophyte development in the TMJ.

## BONE PHENOTYPE

**Figure 3:**
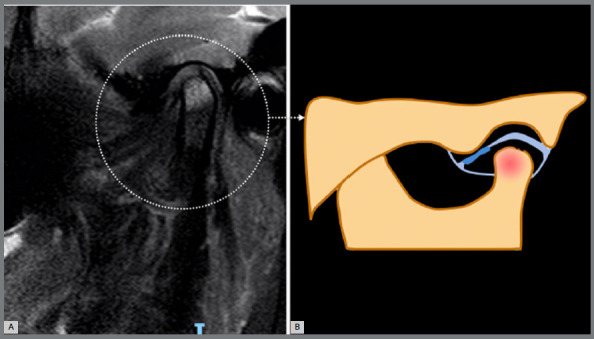
Bone phenotype: a bone marrow lesion in the condyle, and an irregularly condylar head and/or articular fossa contour. A) Referential temporomandibular joint MRI sagittal T2-weighted image. B) Temporomandibular joint scheme. Adapted from ROAMES - Rapid OsteoArthritis MRI Eligibility Score (Source: Roemer et al.^8^, 2020).

The distinctive bone phenotype observed in TMJ OA is characterized by prominent bone marrow lesions (BML), which are discernible through fluid-sensitive fat-suppressed MRI sequences (Fig 3). These non-cystic subchondral areas of ill-defined hyperintensity often accompany cartilage damage, playing a crucial role as predictors of subsequent structural progression and symptom fluctuation in TMJ OA. The complex relationship between the BML pattern in the mandibular condyle and TMJ arthralgia is noteworthy. Notably, the resolution of the edema pattern does not consistently correlate with pain reduction and vice versa,^32^ ^,^ ^33^ suggesting that bone marrow edema in the mandibular condyle does not always contribute to joint pain in patients with intra-articular temporomandibular disorders (TMD). Furthermore, after an improvement in clinical symptoms following arthrocentesis combined with non-surgical treatment, at the meantime interval of around one and a half years, more than 70% of the cases exhibited a persistent bone marrow edema pattern.^33^

Histological evidence indicates that areas of bone marrow edema are almost invariably present in the absence of condylar cortical erosions, potentially representing a distinct disease entity. Most of these marrow alterations are associated with disc displacements, primarily without reduction. However, over time, joints with initial bone marrow abnormalities are likely to develop secondary osteoarthritis.^34^ Thus, the theory of bone mechanotransduction - which refers to the cell’s ability to actively sense, integrate and convert mechanical stimuli into biochemical signals, resulting in intracellular changes, such as the activation of signaling pathways and transcriptional regulation for osteoclastic activity^35^ - appears to be supported by these findings, contrasting with the synovial fluid intrusion hypothesis.^36^

Bone marrow lesions were observed in 30% of the mandibular condyles with cyst-like areas, predominantly located in the central parts of the condyle, with the majority (80%) showing no communication with the joint space,^37^ and its prevalence strongly predict incident subchondral cysts in the same region, even after adjustment for full-thickness cartilage loss, which supports the bone mechanotransduction theory of subchondral cyst formation.^36^ These subchondral cyst-like lesions in the TMJ, particularly in the anterosuperior and central parts of the condyle, are more prevalent on TMJ with MRI images of articular DDwoR.^38^ Despite a tendency for subchondral cysts to naturally resolve over time, the resolution process may manifest as an erosive loss of volume.^39^

Bone sclerosis distribution, characterized by an ill-defined low-signal intensity in the subchondral bone on fluid-sensitive and T1-weighted MRI-images, appears to be ambiguously associated with the presence or absence of erosions.^40^ It is noteworthy that the presence of baseline MRI-detected subchondral sclerosis does not elevate the risk for subsequent cartilage loss in the same knee region. Despite sharing histological features with edema-like BML and likely being connected to mechanical loading, they may represent distinct phases of changes and remodeling in trabecular bone during the loading process. This could elucidate why cohort studies have not demonstrated an increased risk of longitudinal cartilage loss in the same knee region in the presence of subchondral sclerosis. The intriguing question arises as to whether subchondral sclerosis reflects disease inactivity.^41^

Thus, bone marrow MRI-signal intensity holds the potential to serve as a crucial tool for identifying not only the type of BML present, but also the stage of TMJ OA pathology. Gray matter emerges as a potential reference point for evaluating the signal intensity of bone marrow in the mandibular condyle in the same MRI-sequence.^42^ A novel diagnostic approach entails the utilization of the signal intensity ratio (SIR) on MRI to quantitatively assess the quality of condylar bone marrow in the TMJ. This noninvasive approach offers valuable insights into personalized and precise treatment strategies for TMJ degenerative disease. A normal SIR appears to be indicative of a healthy condyle, while a higher or lower SIR may serve as an indicator of different stages of TMJ degenerative disease.^43^ ^,^ ^44^

In summary, the evolving comprehension of the bone phenotype in TMJ OA encompasses a spectrum of manifestations, including BML ranging from edema-like to sclerosis signals, and subchondral cyst-like lesions. These findings not only enhance our understanding of TMJ pathophysiology but also open novel diagnostic avenues for personalized treatment strategies.

## INFLAMMATORY PHENOTYPE

**Figure 4:**
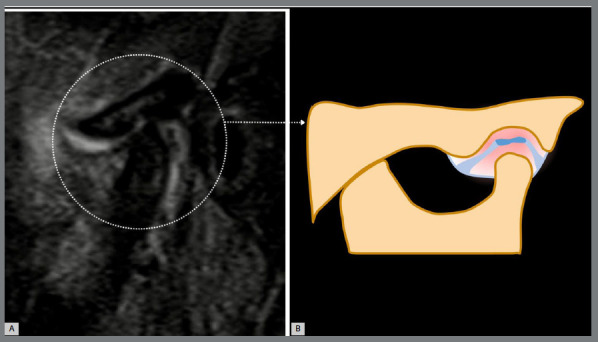
Inflammatory phenotype: a bright signal inside the joint spaces that has a convex configuration in the anterior and/or posterior recesses, and an irregularly condylar head and/or articular fossa contour. A) Referential temporomandibular joint MRI sagittal T2-weighted image. B) Temporomandibular joint scheme. Adapted from ROAMES - Rapid OsteoArthritis MRI Eligibility Score (Source: Roemer et al.^8^, 2020).

From an MRI perspective, the inflammatory phenotype in TMJ OA is characterized by marked synovitis and/or joint effusion, which typically appears as high signal intensity on T2- weighted MRI (Fig 4). In general OA, synovitis is considered a secondary phenomenon linked to cartilage deterioration, and appears to contribute to the progression of cartilage loss. Arthroscopy evaluations have revealed various synovial abnormalities in TMJ OA, with associations between inflammatory synovitis and progressive cartilage damage.^45^

However, although the progression of OA is multifactorial, including biological, mechanical, and psychosocial aspects, a subset of patients shows a dysregulated inflammatory response, characterized by an amplified pro-inflammatory response combined with a lack of attendant anti-inflammatory response. This phenotype has been observed following different forms of intra-articular lesions. Effusion synovitis is significantly greater in those demonstrating a dysregulated inflammatory response, compared to those with a normal response. Additionally, effusion synovitis correlates significantly with synovial fluid concentrations of degradative enzymes and a biomarker of early cartilage degradation.^46^

Intra-articular TMD clinical presentation may vary based on the patient’s age and mandibular condyle bone maturity, influencing its ability to adapt to overload demands. Particularly, in early ages these disorders can often manifest in a highly inflammatory manner. Identifying TMJ involvement in conditions such as juvenile idiopathic arthritis, idiopathic condylar resorption, and other forms of progressive TMJ destruction in children and adolescents can be a challenge in the initial stages, leading to diagnostic confusion. It is crucial to note that determining the appropriate treatment pathway relies heavily on identifying the underlying etiology. Although inflammatory findings on gadolinium-enhanced TMJ MRI can provide valuable information, they alone are insufficient for determining the specific etiology of progressive TMJ destruction. In contrast, characteristics of the TMJ disc and its displacement play a significant role and can serve as crucial differentiators between systemic arthritic and non-arthritic etiologies.^47^

Joint effusion is often observed in MRI, and its diagnostic value for TMJ arthralgia remains unclear. The mean effusion volume should be greater in patients with TMJ OA and arthralgia, but similar in patients with TMJ OA without arthralgia and control subjects.^20^ Spontaneous pain appears to align with MRI findings of joint effusion. However, when considering different types of provoked pain, there is a notable distinction. Pain experienced upon palpation of the masticatory muscles and TMJ does not seem to be associated with MRI findings of joint effusion. In contrast, pain triggered by mandibular opening movement does exhibit a correlation with these MRI findings. This suggests that the relationship between pain symptoms and joint effusion may vary depending on the specific type of pain stimulus, emphasizing the need for a nuanced understanding when assessing and interpreting clinical manifestations in TMJ disorders.^48^

Future research is needed to determine if non-invasive methods, such as MRI or ultrasonography, can accurately identify patients within this pro-inflammatory phenotype and whether this subset is more prone to rapid changes after injury.

## ADAPTIVE PHENOTYPE

A fifth phenotype in adaptive non-osteoarthritic subjects can be identified, comprising individuals with imaging features commonly observed in OA patients, such as flattening and/or subcortical sclerosis. These features are considered indeterminate findings for OA, lacking clinical symptoms or a history indicative of OA. They are typically a normal variation or manifestations of an adaptive response to aging or remodeling due to overload. The presence of this phenotype may not necessitate treatment but rather monitoring, as these imaging features could also serve as precursors to degenerative joint disease.^24^

## INDIVIDUALIZED THERAPEUTIC OPTIONS

TMJ OA is recognized as a comprehensive joint pathology, involving processes such as cartilage degradation, synovial inflammation, and bone remodeling. These intricate mechanisms operate at varying intensities during different stages of the disease, and are currently identified as viable therapeutic targets. Despite structural damage affecting all these tissues and perpetuating the active disease state, a predominant focus in clinical trials related to TMJ OA remains on alleviating pain and/or improving mandibular function. Consequently, there exists a notable gap in evidence regarding the regenerative capabilities of the affected tissues. The prevailing challenge is the development of evidence-based personalized treatments tailored to address the diverse needs of these patients.

**Figure 5:**
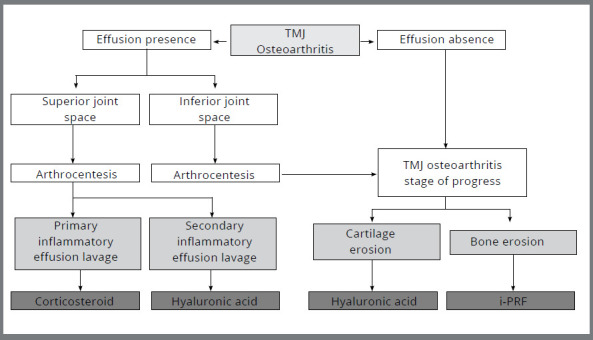
Proposed flow chart for intra-articular injection therapy: Once TMJ OA diagnosis is confirmed, through patient history, physical examination, and MRI images, the presence or absence of joint effusion will determine the need for lavage and anti-inflammatory medications. In cases where joint effusion is absent, an assessment of the TMJ OA stage of progress should be conducted. If only cartilage erosion or both cartilage and bone erosion are observed, hyaluronic acid or injectable platelet-rich fibrin should be selected, respectively. If joint effusion is present, the location of the signal on MRI (i.e., in the superior or inferior joint spaces) will determine the space subjected to joint lavage under arthrocentesis. Following superior joint space arthrocentesis, the selection of intra-articular injection therapy will depend on the presence of primary inflammatory effusion (inflammatory phenotype) or secondary inflammatory effusion (other phenotypes), with corticosteroid or hyaluronic acid being chosen, respectively. In cases where joint effusion is observed in the inferior joint spaces of the TMJ, after arthrocentesis, intra-articular therapy should be selected based on the TMJ OA stage of progress, as previously described.

In the presence of an active inflammatory phenotype in TMJ OA, the preferred approach involves lavage to eliminate primary inflammatory effusion, coupled with the injection of anti-inflammatory drugs like corticosteroids (Fig 5). Arthrocentesis, a minimally invasive procedure for lysis and lavage of the joints, has proven to reduce TMJ arthralgia in the long term,^49^ while maintaining similar mandibular function results as those obtained with non-surgical and more conservative approaches.^50^ A meta-analysis suggested that arthrocentesis performed within three months of conservative treatment might yield additional beneficial results.^51^

Pain control during disease flares should primarily be managed with short-term oral NSAIDs, not exceeding one week regime, to mitigate potential severe adverse effects. Ibuprofen was shown to have a significant pain-reducing effect and increased mandibular function.^52^

When the erosive process initiates and remains at the cartilage level, the primary oral intervention aims at chondroprotection to enhance the self-repair capability of the cartilage. This involves stimulating the proliferation and differentiation of fibrocartilage chondroprogenitors for the formation of new matrix and tissue regeneration.^53^ Although there is still very low evidence regarding therapeutical effects of glucosamine sulfate on TMJ OA, considering a short-term follow-up (12 weeks), glucosamine was as effective as ibuprofen taken two or three times a day,^54^ with the ability to decrease inflammatory biomarkers in synovial fluid.^55^ The most crucial aspect affecting the clinical efficacy of oral glucosamine in the treatment of TMJ OA was the total administration time. Administration of oral glucosamine for a longer period, i.e., 3 months, led to a significant reduction in TMJ pain and a significant increase in maximum mouth opening.^56^

As the superficial layers of fibrocartilage interact with synovial fluid, primarily composed of hyaluronic acid (HA), viscosupplementation with HA is recommended following lysis and lavage, to remove reactive inflammatory mediators. An umbrella review revealed a reduction in pain intensity and improvement in functioning among patients affected by intra-articular TMD after HA injections.^57^ Since this secondary inflammatory process is a consequence of cartilage breakdown, achieving long-term and safe control of the immune reaction to new epitopes, composed of collagen and aggrecan fragments,^58^ can be facilitated through drugs employing oral tolerance approaches.^59^ These approaches have the potential to be beneficial for both cartilage phenotypes, whether erosive atrophic or hypertrophic, particularly in their early stages. However, to date, there is no clinical evidence of their application in the TMJ.

When tissue damage extends to the subchondral bone, regardless of the primary origin of degenerative processes -whether bone or cartilage erosive phenotypes-, and the loss of fibrocartilage coverage becomes evident, the primary therapeutic focus shifts to bone recorticalization. While the removal of inflammatory mediators is crucial to initiate the self-repair process, promising alternatives involve intra-articular injections of biological products with osteogenic properties, such as platelet concentrates, initially platelet-rich plasma (PRP),^60^ and more recently injectable platelet-rich fibrin (i-PRF).^61^ Although these alternatives have been under investigation in the TMJ OA field for almost a decade, only very recently a clinical trial has tested the efficacy of PRP intra-articular inferior joint space injection, an approach previously used only for hyaluronic acid (HA) or dextrose injections.^62^ Compared with HA, better results in imaging analysis in the PRP group were obtained.^63^ In cases where MRI reveals a bone phenotype degenerative process, marked by evident BML but without cortical erosions, controlling the early stages of the process becomes a genuine challenge due to the inaccessibility to the disease niche -the subchondral bone. While attempts to reach the subchondral bone for therapy deposition through intraosseous PRP injections are under investigation in the knee,^64^ there is currently a lack of published information regarding similar initiatives in the TMJ. Synthesis of drug therapy proposed to each TMJ OA stage is shown in Table 2.

**Table 2:** Drug therapy proposal according to each TMJ OA stage.

Phenotype	Oral drugs	Intra-articular injection
Adaptive	x	X
Cartilage erosive	Glucosamine + Chondroitin	Hyaluronic acid
Bone erosion	x	I-PRF
Secondary inflammation	Undenatured Type II Collagen	Hyaluronic acid
Primary effusion	NSAIDs	Corticosteroids

I-PRF = injectable platelet rich fibrin; NSAIDs =Non-steroidal anti-inflammatory drugs.

## FUTURE DIRECTIONS

There is a compelling requirement for consensus-based definitions and recommendations in the realm of OA phenotype research, considering the current knowledge regarding structural phenotypes. The investigation into personalized treatment recommendations for TMJ OA patients is imperative, given the inherent heterogeneity of the disease. The essential exploration of combining clinical, imaging, and biochemical characteristics holds significant promise in refining OA phenotypes. This approach is crucial for fostering a comprehensive understanding and effective management of TMJ OA.

## CONCLUSIONS

In conclusion, the present exploration of MRI-based structural phenotypes in TMJ OA highlights the complexity of the condition. The identification of distinct phenotypes, such as Cartilage Erosive, Hypertrophic, Bone, and Inflammatory Phenotypes, offers valuable insights for personalized therapeutic interventions. Proposed tools like the ROAMES-TMJ (Rapid OsteoArthritis MRI Eligibility Score for TMJ) aim to categorize individuals into specific structural phenotypes, paving the way for tailored treatments. The present findings underscore the need for individualized treatment approaches ranging from very reversible and conservative treatment options to the selective intra-articular injections of different pharmacological or biological products, with anti-inflammatory and/or regenerative properties. As we embrace these insights, consensus-based definitions and recommendations provide a foundation for future research, emphasizing the integration of clinical, imaging, and biochemical data to refine our understanding and enhance personalized management strategies for TMJ OA.

## References

[1] GBD 2021 Osteoarthritis Collaborators (1990).

[2] Indelli PF, Giuntoli M (2018). Early osteoarthritis of the knee from conservative to surgical management. Ann Transl Med.

[3] Andersson MLE, Haglund E, Aili K, Bremander A, Bergman S (2022). Cohort profile the Halland osteoarthritis (HALLOA) cohort-from knee pain to osteoarthritis: a longitudinal observational study in Sweden. BMJ Open.

[4] Chang AH, Almagor O, Lee JJ, Song J, Muhammad LN, Chmiel JS (2023). The natural history of knee osteoarthritis pain experience and risk profiles. J Pain.

[5] Loeser RF, Goldring SR, Scanzello CR, Goldring MB (2012). Osteoarthritis a disease of the joint as an organ. Arthritis Rheum.

[6] Lawrence RC, Felson DT, Helmick CG, Arnold LM, Choi H, Deyo RA (2008). Estimates of the prevalence of arthritis and other rheumatic conditions in the United States Part II. Arthritis Rheum.

[7] Bielajew BJ, Donahue RP, Gabriela Espinosa M, Arzi B, Wang D, Hatcher DC (2021). Knee orthopedics as a template for the temporomandibular joint. Cell Rep Med.

[8] Roemer FW, Collins J, Kwoh CK, Hannon MJ, Neogi T, Felson DT (2020). MRI-based screening for structural definition of eligibility in clinical DMOAD trials Rapid OsteoArthritis MRI Eligibility Score (ROAMES). Osteoarthritis Cartilage.

[9] Roemer FW, Jarraya M, Collins JE, Kwoh CK, Hayashi D, Hunter DJ (2023). Structural phenotypes of knee osteoarthritis potential clinical and research relevance. Skeletal Radiol.

[10] Skármeta NP, Katzmann Rider G, Heir GM (2021). Understanding the phenotypical representations of temporomandibular osteoarthritis for effective management. J Oral Rehabil.

[11] Embree M, Ono M, Kilts T, Walker D, Langguth J, Mao J (2011). Role of subchondral bone during early-stage experimental TMJ osteoarthritis. J Dent Res.

[12] Wang XD, Zhang JN, Gan YH, Zhou YH (2015). Current understanding of pathogenesis and treatment of TMJ osteoarthritis. J Dent Res.

[13] Peck CC, Goulet JP, Lobbezoo F, Schiffman EL, Alstergren P, Anderson GC (2014). Expanding the taxonomy of the diagnostic criteria for temporomandibular disorders. J Oral Rehabil.

[14] Li L, Shi H, Xie H, Wang L (2018). MRI assessment and histopathologic evaluation of subchondral bone remodeling in temporomandibular joint osteoarthritis a retrospective study. Oral Surg Oral Med Oral Pathol Oral Radiol.

[15] Guermazi A, Roemer FW, Jarraya M, Hayashi D (2023). A call for screening MRI as a tool for osteoarthritis clinical trials. Skeletal Radiol.

[16] Iturriaga V, Bornhardt T, Velasquez N (2023). Temporomandibular joint review of anatomy and clinical implications. Dent Clin North Am.

[17] Fang L, Ye Y, Tan X, Huang L, He Y (2021). Overloading stress-induced progressive degeneration and self-repair in condylar cartilage. Ann N Y Acad Sci.

[18] Pucci R, Vellone V, Ramieri V, Cascone P, Della Rocca C (2018). Histological findings in TMJ treated with high condilectomy for internal derangement. J Craniomaxillofac Surg.

[19] Emshoff R, Bertram F, Schnabl D, Stigler R, Steinmaßl O, Rudisch A (2016). Condylar erosion in patients with chronic temporomandibular joint arthralgia: a Cone-Beam Computed Tomography study. J Oral Maxillofac Surg.

[20] Xu J, Wang D, Yang C, Wang F, Wang M (2023). Reconstructed magnetic resonance image-based effusion volume assessment for temporomandibular joint arthralgia. J Oral Rehabil.

[21] Bae S, Park MS, Han JW, Kim YJ (2017). Correlation between pain and degenerative bony changes on cone-beam computed tomography images of temporomandibular joints. Maxillofac Plast Reconstr Surg.

[22] Abrahamsson AK, Arvidsson LZ, Småstuen MC, Larheim TA (2020). Improvement of bone erosive temporomandibular joint (TMJ) abnormalities in adolescents undergoing nonsurgical treatment a longitudinal study. Dentomaxillofac Radiol.

[23] Sun CK, Li YB, Ma HS, Li G, Sun ZP, Sun LS (2023). Natural course of severe temporomandibular joint osteoarthrosis evaluated by a novel condylar remodelling scoring system and quantitative volumetric analysis. Int J Oral Maxillofac Surg.

[24] Ahmad M, Schiffman EL (2016). Temporomandibular joint disorders and orofacial pain. Dent Clin North Am.

[25] Junker S, Krumbholz G, Frommer KW, Rehart S, Steinmeyer J, Rickert M (2016). Differentiation of osteophyte types in osteoarthritis proposal of a histological classification. Joint Bone Spine.

[26] Sahoo NK, Mowar A, Pandey S, Vashisht S, Vaswani H (2023). Histopathological Interpretation of TMJ Osteophyte report and review. J Maxillofac Oral Surg.

[27] Ottersen MK, Larheim TA, Hove LH, Arvidsson LZ (2023). Imaging signs of temporomandibular joint osteoarthritis in an urban population of 65-year-olds a cone beam computed tomography study. J Oral Rehabil.

[28] Roelofs AJ, Kania K, Rafipay AJ, Sambale M, Kuwahara ST, Collins FL (2020). Identification of the skeletal progenitor cells forming osteophytes in osteoarthritis. Ann Rheum Dis.

[29] Venne G, Tse MY, Pang SC, Ellis RE (2020). Mechanically-induced osteophyte in the rat knee. Osteoarthritis Cartilage.

[30] Grossmann E, Remedi MP, Ferreira LA, Carvalho ACP (2016). Magnetic resonance image evaluation of temporomandibular joint osteophytes influence of clinical factors and artrogenics changes. J Craniofac Surg.

[31] Dias IM, Cordeiro PCF, Devito KL, Tavares MLF, Leite ICG, Tesch RS (2016). Evaluation of temporomandibular joint disc displacement as a risk factor for osteoarthrosis. Int J Oral Maxillofac Surg.

[32] Higuchi K, Chiba M, Sai Y, Yamaguchi Y, Nogami S, Yamauchi K (2020). Relationship between temporomandibular joint pain and magnetic resonance imaging findings in patients with temporomandibular joint disorders. Int J Oral Maxillofac Surg.

[33] Chiba M, Kumagai M, Fukui N, Echigo S (2006). The relationship of bone marrow edema pattern in the mandibular condyle with joint pain in patients with temporomandibular joint disorders longitudinal study with MR imaging. Int J Oral Maxillofac Surg.

[34] Sano T, Westesson PL, Larheim TA, Rubin SJ, Tallents RH (1999). Osteoarthritis and abnormal bone marrow of the mandibular condyle. Oral Surg Oral Med Oral Pathol Oral Radiol Endod.

[35] Zhao Z, Li Y, Wang M, Zhao S, Zhao Z, Fang J (2020). Mechanotransduction pathways in the regulation of cartilage chondrocyte homoeostasis. J Cell Mol Med.

[36] Crema MD, Roemer FW, Zhu Y, Marra MD, Niu J, Zhang Y (2010). Subchondral cystlike lesions develop longitudinally in areas of bone marrow edema-like lesions in patients with or at risk for knee osteoarthritis detection with MR imaging--the MOST study. Radiology.

[37] Nozawa M, Ogi N, Ariji Y, Kise Y, Nakayama M, Nishiyama M (2020). Reliability of diagnostic imaging for degenerative diseases with osseous changes in the temporomandibular joint with special emphasis on subchondral cyst. Oral Radiol.

[38] Souza-Pinto GN, Herreira-Ferreira M, Grossmann E, Brasil DM, Hara GF, Groppo FC (2023). Assessment of temporomandibular joint bone changes associated with anterior disc displacement an MRI cross-sectional study. J Stomatol Oral Maxillofac Surg.

[39] Takaoka R, Koishi Y, Kuyama K, Ueda Y, Ishigaki S, Uchiyama Y (2023). Cross-sectional and longitudinal assessment of subchondral cysts in temporomandibular joints clinical and MRI study with a mean follow-up of 66 months. J Prosthodont Res.

[40] Larheim TA, Westesson PL, Hicks DG, Eriksson L, Brown DA (1999). Osteonecrosis of the temporomandibular joint correlation of magnetic resonance imaging and histology. J Oral Maxillofac Surg.

[41] Crema MD, Cibere J, Sayre EC, Roemer FW, Wong H, Thorne A (2014). The relationship between subchondral sclerosis detected with MRI and cartilage loss in a cohort of subjects with knee pain the knee osteoarthritis progression (KOAP) study. Osteoarthritis Cartilage.

[42] Yamamoto A, Sano T, Otonari-Yamamoto M, Nishikawa K, Kwok E (2008). A potential reference point for assessment of condylar bone marrow of the temporomandibular joint on proton density weighted images. Cranio.

[43] Wan S, Sun Q, Xie Q, Dong M, Liu Z, Yang C (2023). The retrospective study of magnetic resonance imaging signal intensity ratio in the quantitative diagnosis of temporomandibular condylar resorption in young female patients. J Pers Med.

[44] Yajima A, Sano T, Otonari-Yamamoto M, Otonari T, Ohkubo M, Harada T (2007). MR evidence of characteristics in symptomatic osteoarthritis of the temporomandibular joint increased signal intensity ratio on proton density-weighted images of bone marrow in the mandibular condyle. Cranio.

[45] Celotti C, Martín-Granizo R, De La Sen Ó (2022). Correlation of arthroscopic findings with clinical-radiological signs and symptoms of temporomandibular joint dysfunction retrospective study of 829 joints. Int J Oral Maxillofac Surg.

[46] Jacobs CA, Stone AV, Conley CEW, Abed V, Huebner JL, Kraus VB (2023). Increased effusion synovitis for those with a dysregulated inflammatory response after an anterior cruciate ligament injury. Cureus.

[47] Bousquet B, Kellenberger CJ, Caprio RM, Jindal S, Resnick CM (2023). Does magnetic resonance imaging distinguish juvenile idiopathic arthritis from other causes of 27 progressive temporomandibular joint destruction. J Oral Maxillofac Surg.

[48] Park HN, Kim KA, Koh KJ (2014). Relationship between pain and effusion on magnetic resonance imaging in temporomandibular disorder patients. Imaging Sci Dent.

[49] Guarda-Nardini L, Meneghini M, Zegdene S, Manfredini D (2021). Temporomandibular joint arthrocentesis in patients with degenerative joint disease a 10- to 22-year follow-up. J Oral Facial Pain Headache.

[50] Tang YH, Vos LM, Tuin AJ, Huddleston Slater JJR, Gareb B, van Bakelen NB (2023). Arthrocentesis versus non-surgical intervention as initial treatment for temporomandibular joint arthralgia a randomized controlled trial with long-term followup. Int J Oral Maxillofac Surg.

[51] Li DTS, Wong NSM, Li SKY, McGrath CP, Leung YY (2021). Timing of arthrocentesis in the management of temporomandibular disorders an integrative review and meta-analysis. Int J Oral Maxillofac Surg.

[52] Christidis N, Al-Moraissi EA, Barjandi G, Svedenlöf J, Jasim H, Christidis M (2024). Pharmacological treatments of temporomandibular disorders a systematic review including a network meta-analysis. Drugs.

[53] Artuzi FE, Puricelli E, Baraldi CE, Quevedo AS, Ponzoni D (2020). Reduction of osteoarthritis severity in the temporomandibular joint of rabbits treated with chondroitin sulfate and glucosamine. PLoS One.

[54] Melo G, Casett E, Stuginski-Barbosa J, Guerra ENS, Fernandes DA, Porporatti AL (2018). Effects of glucosamine supplements on painful temporomandibular joint osteoarthritis a systematic review. J Oral Rehabil.

[55] Ruiz-Romero V, Toledano-Serrabona J, Gay-Escoda C (2022). Efficacy of the use of chondroitin sulphate and glucosamine for the treatment of temporomandibular joint dysfunction a systematic review and meta-analysis. Cranio.

[56] Derwich M, Górski B, Amm E, Pawlowska E (2023). Oral glucosamine in the treatment of temporomandibular joint osteoarthritis a systematic review. Int J Mol Sci.

[57] Agostini F, Ferrillo M, Bernetti A, Finamore N, Mangone M, Giudice A (2023). Hyaluronic acid injections for pain relief and functional improvement in patients with temporomandibular disorders An umbrella review of systematic reviews. J Oral Rehabil.

[58] Bay-Jensen AC, Mobasheri A, Thudium CS, Kraus VB, Karsdal MA (2022). Blood and urine biomarkers in osteoarthritis - an update on cartilage associated type II collagen and aggrecan markers. Curr Opin Rheumatol.

[59] Lugo JP, Saiyed ZM, Lane NE (2016). Efficacy and tolerability of an undenatured type II collagen supplement in modulating knee osteoarthritis symptoms a multicenter randomized, double-blind, placebo-controlled study. Nutr J.

[60] Haddad C, Zoghbi A, El Skaff E, Touma J (2023). Platelet-rich plasma injections for the treatment of temporomandibular joint disorders a systematic review. J Oral Rehabil.

[61] Isik G, Kenç S, Özveri Koyuncu B, Günbay S, Günbay T (2022). Injectable platelet-rich fibrin as treatment for temporomandibular joint osteoarthritis a randomized controlled clinical trial. J Craniomaxillofac Surg.

[62] De Nordenflycht D, Ayala A, Orellana L, Tesch RS (2023). Intra-articular injections in the TMJ inferior joint space a scoping review. J Oral Rehabil.

[63] Liu SS, Xu LL, Liu LK, Lu SJ, Cai B (2023). Platelet-rich plasma therapy for emporomandibular joint osteoarthritis a randomized controlled trial. J Craniomaxillofac Surg.

[64] Barman A, Bandyopadhyay D, Mohakud S, Sahoo J, Maiti R, Mukherjee S (2023). Comparison of clinical outcome, cartilage turnover, and inflammatory activity following either intra-articular or a combination of intra-articular with intra-osseous platelet-rich plasma injections in osteoarthritis knee a randomized, clinical trial. Injury.

